# Gating of a pH-Sensitive K_2P_ Potassium Channel by an Electrostatic Effect of Basic Sensor Residues on the Selectivity Filter

**DOI:** 10.1371/journal.pone.0016141

**Published:** 2011-01-25

**Authors:** Leandro Zúñiga, Valeria Márquez, Fernando D. González-Nilo, Christophe Chipot, L. Pablo Cid, Francisco V. Sepúlveda, María Isabel Niemeyer

**Affiliations:** 1 Centro de Estudios Científicos (CECS), Valdivia, Chile; 2 Centro de Ingeniería de la Innovación del CECS (CIN), Valdivia, Chile; 3 Centro de Bioinformática y Simulación Molecular, Universidad de Talca, Talca, Chile; 4 Beckman Institute, University of Illinois at Urbana-Champaign, Urbana, Illinois, United States of America; 5 Équipe de Dynamique des Assemblages Membranaires, Unité Mixte de Recherche CNRS/UHP 7565, Université de Nancy, Vandoeuvre-lès-Nancy, France; University of Queensland, Australia

## Abstract

K^+^ channels share common selectivity characteristics but exhibit a wide diversity in how they are gated open. Leak K_2P_ K^+^ channels TASK-2, TALK-1 and TALK-2 are gated open by extracellular alkalinization. The mechanism for this alkalinization-dependent gating has been proposed to be the neutralization of the side chain of a single arginine (lysine in TALK-2) residue near the pore of TASK-2, which occurs with the unusual p*K_a_* of 8.0. We now corroborate this hypothesis by transplanting the TASK-2 extracellular pH (pH_o_) sensor in the background of a pH_o_-insensitive TASK-3 channel, which leads to the restitution of pH_o_-gating. Using a concatenated channel approach, we also demonstrate that for TASK-2 to open, pH_o_ sensors must be neutralized in each of the two subunits forming these dimeric channels with no apparent cross-talk between the sensors. These results are consistent with adaptive biasing force analysis of K^+^ permeation using a model selectivity filter in wild-type and mutated channels. The underlying free-energy profiles confirm that either a doubly or a singly charged pH_o_ sensor is sufficient to abolish ion flow. Atomic detail of the associated mechanism reveals that, rather than a collapse of the pore, as proposed for other K_2P_ channels gated at the selectivity filter, an increased height of the energetic barriers for ion translocation accounts for channel blockade at acid pH_o_. Our data, therefore, strongly suggest that a cycle of protonation/deprotonation of pH_o_-sensing arginine 224 side chain gates the TASK-2 channel by electrostatically tuning the conformational stability of its selectivity filter.

## Introduction

Potassium channels are membrane proteins that catalyse the permeation of K^+^ ions across the plasma membrane down their electrochemical gradient. Generally, they consist of six membrane-spanning α-helices and a highly conserved P-domain, which forms the selectivity filter. The P-domain and two of the transmembrane α-helices form the K^+^ channel pore in a tetrameric symmetric arrangement [1]. K_2P_ (also known as KCNK) K^+^ channels [2]–[4], of which there are sixteen mammalian members, have of two P-domains and four α-helices in each subunit and it has been expected that two subunits would be necessary to form a functional structure. Biochemical and mutagenesis studies support this arrangement [5], [6]. More recently a detailed mutational analysis and continuum electrostatic free energy calculations has strongly supported a model for K_2P_ channels with each subunit contributing two P-domains to a pore, with identical P-domains facing each other through the pore's centre to form a complex with bilateral symmetry and an ion conduction pathway with pseudo-4-fold symmetry [7]. Unlike other K^+^ channels, K_2P_ channels are generally open at resting potential and constitute therefore K^+^-selective leaks that are fundamental to the maintenance of the resting potential and the function of various cells including those of nerves, muscles and epithelia. Although they underlie the leak conductance of most cells, K_2P_ channels do not lack gating, and are exquisitely regulated by free fatty acids, membrane tension, G-proteins, and pH among other variables [2]–[4].

Among K_2P_ channels gated by extracellular pH, TASK-1 and TASK-3 form a part of the TASK subfamily and are blocked by extracellular protons [5], [8]–[10]. TREK-1 and TREK-2 are respectively inhibited and activated by extracellular acidification [11], [12]. A third group (TALK) of K_2P_ channels comprises TASK-2, TALK-1 and TALK-2, and are activated by extracellular alkalinization. TASK-2 has been shown to be important in the regulation of cell volume [13], [14] and its physiological role has been surmised from studies in TASK-2 knockout mice [15] that present metabolic acidosis and hypotension secondary to renal loss of HCO^-^_3_. It is thought that transport of HCO^−^_3_ in the proximal tubule is coupled to TASK-2 activity through extracellular alkalinization, and genetic ablation of TASK-2 leads to a proximal renal tubular acidosis-like syndrome. TALK-1 and -2 are activated by strong extracellular alkalinization and are highly expressed in the pancreas [16], a site of intense HCO^−^_3_ secretion. The knockout mouse has also shown that TASK-2 channels expressed in retrotrapezoid nucleus neurons are important in mediating central CO_2_ and O_2_ chemosensitivity [17]. TASK-2 channels are hypothesized to maintain a hyperpolarized condition of retrotrapezoid nucleus neurons, thereby preventing a respiratory increase at low CO_2_ partial pressure [17]. The strong ventilatory drive at high partial pressure of CO_2_ could be the consequence of a decrease in TASK-2 channel activity that might be mediated by the recently uncovered intracellular pH-sensitivity of TASK-2 [18].

It was proposed that a group of four lysine and one glutamic-acid residues located in the extracellular loop between TM1 and P1 act in a concerted fashion as the sensor controlling the pH_o_-gating of TASK-2 [19]. More recently, we challenged this view and endeavored to use molecular simulations and site-directed mutagenesis experiments to demonstrate that the pH_o_-sensor mediating the gating of TASK-2 is an arginine (R224) residue sited towards the extracellular end of TM4 [20]. The hypothesis that R224 is the sensor was supported by the loss of pH_o_-sensitivity after its neutralization, and by the modulation of the pH_o_-sensitivity upon replacement with amino acids of different p*K_a_* values, or alterations in its environment. Modulation of TASK-2 by extracellular pH involves changes in open probability (P_o_) without affecting single-channel conductance [9], [21]. As to the molecular mechanism for modulation, we reasoned that in the protonated form, R224 prevents occupancy of the selectivity filter by K^+^, thus creating a blocked state, a situation relieved by neutralization of R224 at alkaline pH [20], [22]. As discussed in a recent comprehensive review [23], H^+^-dependent gating at the selectivity filter seems to be a frequently encountered feature in K_2P_ channels.

In the present contribution, we address three questions about pH_o_-sensing in these channels: First, we validate the previously identified role of R224 as the pH_o_-sensor of TASK-2 by transferring pH_o_-sensing into a TASK-3 channel that had been rendered pH_o_-insensitive by mutation. Second, we use a concatenated channel approach to show that both TASK-2 arginine pH_o_ sensors of this dimeric channel must be neutralized for channel opening, without mutual interaction. Third and last, we show that the action of a charged R224 pH_o_-sensing residue occurs through an electrostatic effect that enhances the flexibility of the selectivity filter and increases the height of energy barriers between binding sites.

## Results

### Transplantation of TASK-2 pH_o_-sensor into the background of a pH_o_-insensitive TASK-3 channel transfers pH_o_-dependent pore gating

**Figure 1:**
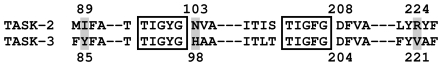
Aligment of pHo-sesnor regions of TASK-2 and TASK-3 channels. The amino acid sequences of the regions involved in extracellular [H^+^] gating for these two K_2P_ channels are shown. Highlighted in gray are the pH_o_-sensors R224 and H98 of TASK-2 and TASK-3 respectively, and also TASK-2 I89 that corresponds to the envioronment of R224. The signature sequences for the pore regions P1 and P2, left and right, are boxed.

TASK-3 is a K_2P_ K^+^ channel that is open at physiological pH_o_, but is closed by extracellular acidification. The pH_o_-sensor in TASK-3 has been identified as a histidine residue (H98) located immediately extracellular to the selectivity filter [5], [9]. The position equivalent to H98 of TASK-3 is occupied by an asparagine residue (N103) in TASK-2, whilst V221 occupies the position homologous to pH_o_-sensing R224 of TASK-2 (Figure 1).

**Figure 2:**
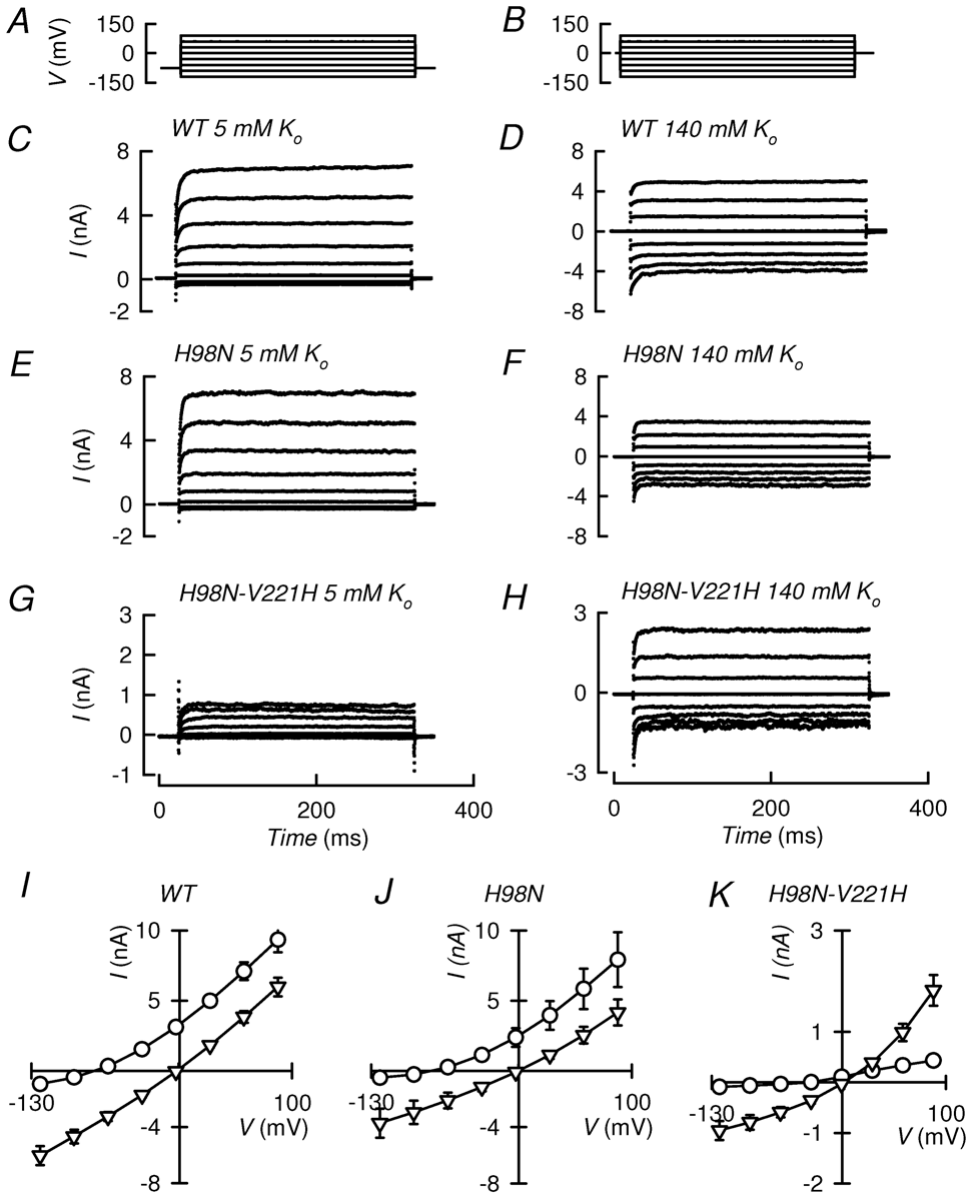
Transplanting a TASK-2-type pH_o_-sensor onto the pH_o_-insensitive TASK-3 channel mutant: effect on rectification, time-dependence and selectivity. Currents recorded were elicited by transfection of WT TASK-3, TASK-3-H98N or TASK-3-H98N-V221H into HEK-293 cells. Measurements were done in the whole-cell recording mode of the patch-clamp technique, using the voltage protocols in A (for currents shown in C, E and G) and B (for D, F and H). The intracellular solution contained 140 mM K^+^. Results in C and D correspond to TASK-3 (WT) and those in E and F and G and H to TASK-3-H98N and TASK-3-H98N-V221H respectively. In C, E and G the extracellular medium had 135 mM Na^+^ and 5 mM K^+^. In D, F and H, extracellular Na^+^ was replaced by an equimolar amount of K^+^. I, J and K show current-voltage relations for WT TASK-3and its mutants under the different extracellular K^+^ concentrations (circles 5 mM; triangles 140 mM). Results are means ± SEM. Number of experiments were respectively for 5 and 140 mM: 6 and 8 for WT TASK-3, 6 and 6 for TASK-3-H98N and 6 and 8 for TASK-3-H98N-V221H.

**Figure 3:**
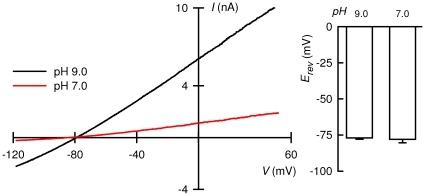
Constancy of apparent K^+^/Na^+^ selectivity in TASK-2 after inhibition by extracellular acidification. Current-voltage relation for a cell expressing TASK-2 at pH 9.0 and 7.0, chosen to yield respectively ∼90 and ∼15% of the maximal activation as judged from complete activity vs. pH curves [20]. The intracellular solution contained 140 mM K^+^ and extracellular bath had 135 mM Na^+^ and 5 mM K^+^. The current-voltage relations were taken from 250 ms-voltage ramps given from the most hyperpolarised voltage. The graph on the right-hand side summarises average E_rev_ measurements at both pH values in the form of means ± SEMs of 6 experiments. There was no significant difference between E_rev_ as analysed by paired t-test.

We reasoned that introducing a basic amino acid at position 221 in the background of TASK-3-H98N, which is pH_o_-insensitive, should transfer TASK-2-type pH_o_-sensitivity. Figure 2C-F shows a comparison of the currents generated by TASK-3 and the TASK-3-H98N mutant in physiological and high-K^+^ concentrations. The currents were qualitatively similar in WT and mutant channel and, as seen in current-voltage relations in Figure 2I and J, Na^+^/K^+^ selectivity was not altered. Transplantation of a TASK-2-type sensor in the background of the pH_o_-insensitive TASK-3-H98N channel was done by mutating residue V221 to H. Histidine was chosen, rather than the much more basic arginine present in TASK-2, because it should allow us to work at less extreme pH values given that TASK-3 lacks the hydrophobic environment thought to lower the p*K_a_* of the sensing arginine of TASK-2 [20]. Although TASK-3-H98N-V221H double mutant could be readily expressed in HEK-293 cells, it had low currents and poor selectivity towards K^+^ over Na^+^ when assayed at 5 mM extracellular K^+^. Robust currents could, however, be restored using 140 mM extracellular K^+^ (Figure 2G-H and K). Loss of selectivity has been associated with gate closure occurring at the outer part of the pore in K_2P_ channels and is thought to occur by what has been termed collapse of the selectivity filter [23]. This does not occur upon closure of TASK-2 channels by acidification as can be seen in Figure 3, where no significant change in reversal potential, and hence selectivity, took place upon switching between pH 9.0 and 7.0. These pH values were chosen to yield respectively ∼90 and ∼15% of the maximal activation. An equivalent experiment performed with TASK-1 or TASK-3 was accompanied by significant apparent selectivity loss as illustrated in Figure S1.

**Figure 4:**
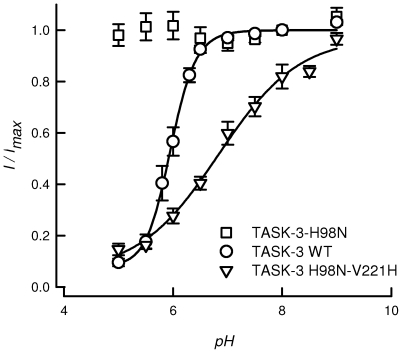
Conferring pH_o_ sensitivity to a TASK-3 insensitive mutant by TASK-2 pH_o_-sensor transplantation. The graph shows extracellular pH-dependence curves of WT TASK-3 (circles, n = 8) and for its mutants TASK-3-H98N (squares, n = 6) and TASK-3-H98N-V221H (triangles, n = 8). Results are shown as means ± SEM. The lines are fits of the Hill equation and were constructed using the average of fitted parameters of the individual experiments.

Transplantation of the TASK-2 type pH_o_-sensor on the background of a pH_o_-insensitive TASK-3 readily transferred the type of sensitivity to pH_o_ seen with TASK-2. Figure 4 shows that the normally pH_o_-insensitive TASK-3-H98N acquired full pH_o_-sensitivity upon further V221H mutation. When assayed in high-K^+^ symmetrical solution, the new double mutant became activatable by alkalinization with a p*K_1/2_* of 6.86±0.11 and *n*_H_ of 0.63±0.06. These results demonstrate that TASK-2-type pH_o_-sensitivity is readily transplanted to TASK-3 channels with their native sensor invalidated. This is consistent with the importance of a charged residue at the position equivalent to R224 of TASK-2 in determining the permeation properties of the channel.

### Role of individual pH_o_-sensors in the gating of TASK-2 K^+^ channels

pH_o_-gating of TASK-2 occurs with a p*K_1/2_* of 8.0, is not cooperative and is mediated by neutralization of arginine 224, so that mutant TASK-2-R224A is pH_o_-independent [20]. To find out whether pH_o_-sensors occurring in both subunits of these homodimeric channels are required in the gating process, we have linked covalently the C-terminus of one channel subunit with the N-terminus of the following to form concatenated structures containing either a normal set of pH_o_-sensors, mixed structures featuring one enabled and one neutralised pH_o_-sensor, or two disabled pH_o_-sensors.

**Figure 5:**
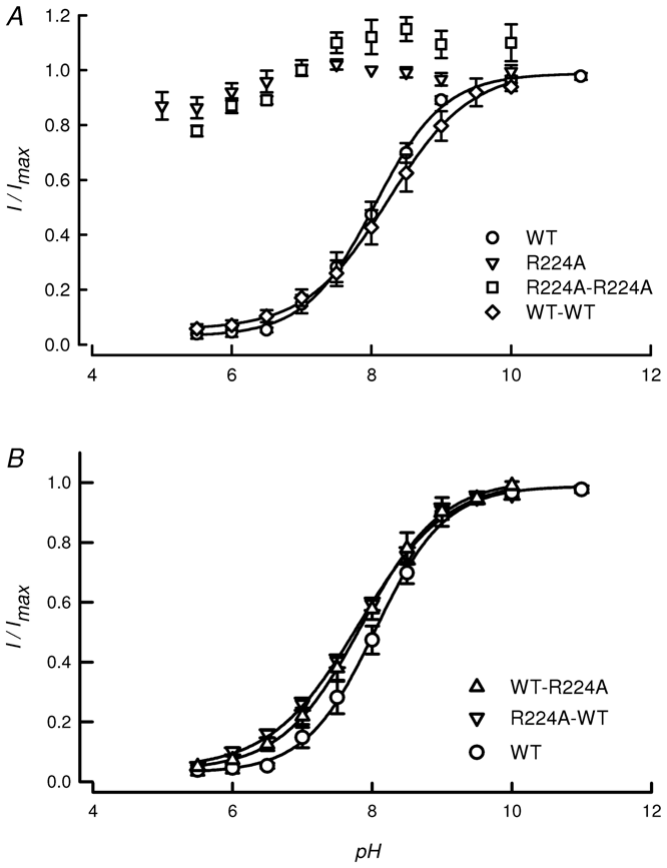
Extracellular pH sensitivity of concatenated constructs of WT and pH_o_-insensitive TASK-2 channels. The graph in A shows extracellular pH-dependence curves of WT TASK-2 (circles) and its mutant TASK-2-R224A (triangles), taken from [20]. Also shown are data for WT-WT (n = 4) and R224A-R224A (n = 6). In B the data for WT TASK-2 monomer are repeated for comparison with the pH-dependence curves of WT-R224A (n = 6) and R224A-WT (n = 6) mixed concatenated constructs. Results are shown as means ± SEM. The lines are fits of the Hill equation constructed using the average of fitted parameters of the individual experiments.

Concatenation did not prevent expression of the channels, which presented normal time dependence for current development, selectivity of K^+^ over Na^+^ and open-channel rectification (Figure S2). Next, we tested their pH_o_-sensitivity. As shown in Figure 5, WT-WT TASK-2 concatenated constructs behaved similarly to the non-concatenated channels with p*K_1/2_* 8.2±0.15, whilst doubly-mutated TASK-2-R224A constructs lacked pH_o_-dependence. The mixed WT-R224A and R224A-WT TASK-2 constructs had pH_o_-dependencies akin to those of WT and WT-WT channels. These data indicate that sensors in both subunits of these dimeric structures must be neutralised in order to open the TASK-2 channels. It also rules out the interaction of TASK-2 pH_o_-sensors located in different subunits.

### Energetics of K^+^ transport across the selectivity filter of TASK-2

**Figure 6:**
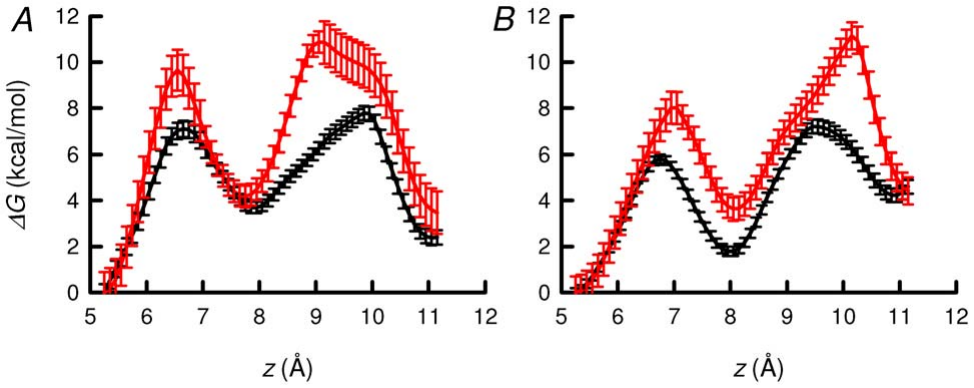
Free energy profile obtained from ABF calculation, delineating the transport of a K^+^ ion across TASK-2 channel permeation pathway. The error bars represent the standard error of the free energy difference at each *z* in the final free energy profile. A, The free energy profile of system with the R224 residues neutralized in both monomers (black line) and the charged-charged situation (red line). B, Free energy profile of system with neutral-R224^0^-A224 state of TASK-2 (black line) and charged R224^+^-A224 state (red line).

**Figure 7:**
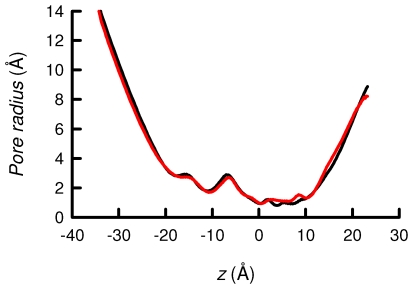
Pore radius profile of TASK-2 models. Pore radius was evaluated along the pore axis (z). The selectivity filter extends from z = 0 to z = 10 Å. Radius profiles for R224^0^-R224^0^ (black line) and R224^+^-R224^+^ (red line) models are shown. The calculation was done using the HOLE algorithm [52] and each profile was obtained as a mean value of all conformations.

In order to gain new insight into the energetics of the permeation of K^+^ ions in the selectivity filter of TASK-2, we have examined the underlying free-energy profiles in this region of the channel employing the adaptive biasing force (ABF) method [24]. This calculation, carried out using an homology model of TASK-2 [20], highlighted the K^+^-binding sites (S_0_, S_1_ and S_2_) in the narrow selectivity filter, which have been hitherto detected in all K^+^ channels examined by X-ray diffraction, from the original KcsA structure to the recently solved Kir 2.2 inward rectifier [25], [26]. Figure 6A shows the free-energy profiles determined for those channels in which the R224 sensors are either in a charged state, or have been neutralised by deprotonation. The channel featuring both R224 sensors charged (Figure 6A, red line) exhibits high free-energy barriers, on the order of 6–8 kcal/mol, both for the S_0_-S_1_ and the S_1_-S_2_ transitions. Neutralisation of both R224 residues (Figure 6A, black line) was accompanied by a decrease of about 4 kcal/mol for the S_0_-S_1_ barrier and more than 2 kcal/mol for S_1_-S_2_. The R224^+^-R224^+^ channel does not exhibit significant alterations in the radius of its selectivity filter, compared to its R224^0^-R224^0^ homologue (Figure 7). Free-energy profile plots are shown in Figure 6B for the model containing one WT, R224-containing monomer and one R224A mutant subunit. The channel featuring one neutral R224 and one A224 residues has an S_0_-S_1_ free-energy barrier about 3 kcal/mol high, increasing by 3.5 kcal/mol upon charging R224. The S_1_-S_2_ barrier amounts in both cases near 4 kcal/mol. The deepest free-energy valley emerges in all cases at S_2_, the site of highest stability for K^+^ coordination.

The free-energy barriers seen under active channel conditions, with neutral R224 sensors in both subunits or under neutral-R224-A224 condition, are in reasonable agreement with those reported previously for KcsA and found to be consistent with a high-rate translocation of the ions along the channel [27]. In those instances where either one or two R224 sensors were charged, the observed S_0_-S_1_ barrier rose to 6–7.5 kcal/mol, and that for S1-S2 to 4–6 kcal/mol. Such an increase is expected to hamper significantly the translocation of the ion along the selectivity filter.

### Van der Waals and Coulombic contributions to the free energy

**Figure 8:**
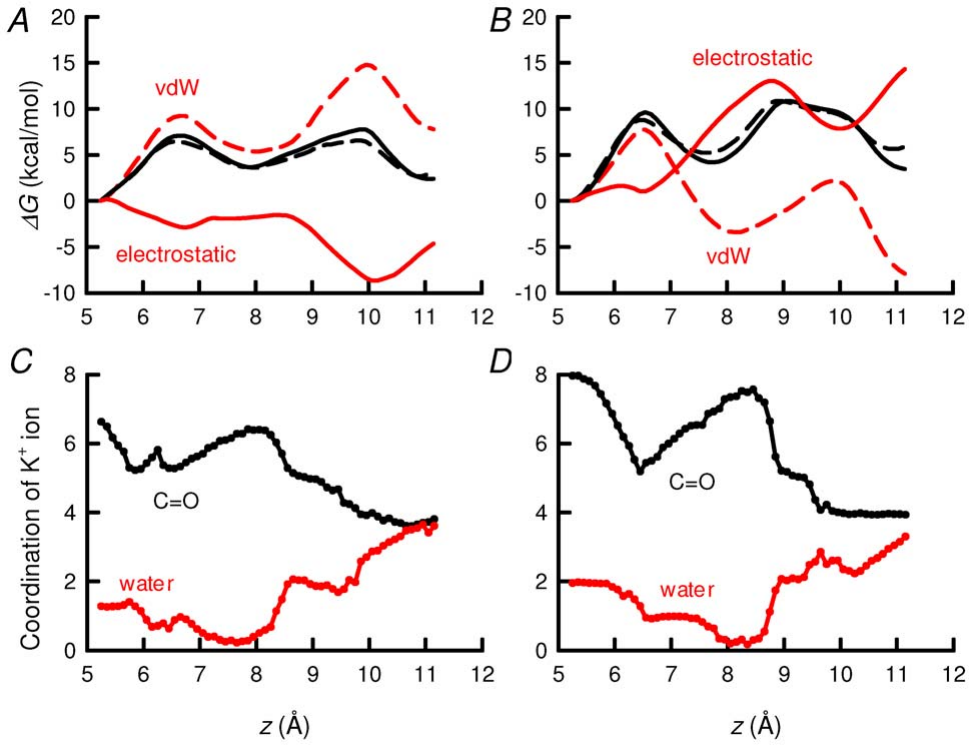
Van der Waals and Coulombic contributions to the free energy. Electrostatic and van der Waals forces (A). Partitioning of the PMF of the system of neutral–neutral state of TASK-2 (solid black line) into vdW energy (dashed red line) and electrostatic energy (solid red line). Black dashed line is the sum of van der Waals and electrostatic contribution. B, Partitioning of PMF of the system of neutral-charged state of TASK-2 (solid black line) into vdW energy (dashed red line) and electrostatic energy (solid red line). Black dashed line is the sum of van der Waals and electrostatic contribution. C, Coordination of K^+^ ion. Solid black line corresponds to number of carbonyl oxygen atoms chelating the ion in the neutral-neutral state. Solid red line corresponds to water oxygens coordinating the ion. D, Coordination of K^+^ ion in charged-charged state of TASK-2. Solid black line represents number of carbonyl oxygens and red line the number of water oxygens coordinating the ion.

Figure 8 depicts a decomposition of the free energy into van der Waals (vdW) and electrostatic contributions for the R224 neutral-neutral (R224^0^-R224^0^) and charged-charged (R224^+^-R224^+^) states of TASK-2. In the R224^0^-R224^0^ system (Figure 8A), the cavity exhibits a negative electrostatic potential favouring ion affinity for the selectivity filter. The selectivity filter, under these premises, is narrow and topologically optimized to facilitate the dehydration of K^+^ ions. This environment is conducive to an increase of the vdW energy as short-range contacts form with the ion translocating from S_0_ to S_1_. The ion being dehydrated before it permeates the selectivity filter, steric hindrances upon entering the narrow cavity are necessarily lessened. At the same time, the electrostatic energy appears to compensate unfavourable vdW interactions. Furthermore, the electrostatic term is favourable along the reaction coordinate, with a free-energy minimum emerging between S_0_ and S_1_. In the R224^0^-R224^0^ channel, vdW repulsions are compensated for by electrostatic contributions rendered favourable through enhanced interaction with the carbonyl moieties of the selectivity filter. Put together, this environment contributes to free-energy barriers compatible with K^+^ translocation.

The R224^+^-R224^+^ channel does not exhibit significant alterations in the radius of its selectivity filter, compared to its R224^0^-R224^0^ homologue (data shown supplementary material). The negative vdW free-energy contribution (Figure 8B) suggests lesser steric hindrance in the former channel than in the latter, which is indicative that the ion forms favourable vdW contacts as it translocates from S_0_ to S_1_. This result is also suggestive that the selectivity filter acquired more flexibility. The presence of the two positively charged sensing arginine residues generates a strong electrostatic repulsion that is not compensated by the short-range interaction of the K^+^ ion and the oxygen atoms of the selectivity filter.

The K^+^ ion has a first hidration sphere with a coordination number of 8. As the ion enters the selectivity filter these water molecules are progressively replaced by the oxygens of the backbone of the selectivity filter GYGD/N motif. We compare below the solvation number of K^+^ ions within the selectivity filter of TASK-2 in the R224^+^-R224^+^ and R224^0^-R224^0^ conditions. As can be seen from a comparison between Figures 8C and D, high electrostatic energy and favourable vdW interactions in the R224^+^-R224^+^ channel are accompanied by a greater number of carbonyl oxygen atoms chelating the ion. The greater chelation of the permeant ion is evident at S_1_ (and also at S_2_), where the coordination number reaches 8 for carbonyl groups (10 at S_2_, with 2 coordinated water molecules). This result ought to be compared to a carbonyl coordination number of 6 at S_1_ (and S_2_) in the R224^0^-R224^0^ channel. Strong binding at S_1_ and S_2_ in the R224^+^-R224^+^ channel accounts for the high free-energy barriers associated to ion translocation in the selectivity filter, thus providing a mechanistic insight into pore gating modulated by the charge state of the sensing R224 residues.

## Discussion

The molecular mechanisms of K^+^ channel gating have been widely studied functionally and also been surmised from structural atomic-level studies [28]. Three different gating modes have been uncovered. The first was identified by comparing KcsA and MthK structures in the closed and open conformations respectively [1], [29]. These structures differ in the position of the inner helices lining the conduction pathway intracellular to the selectivity filter. The four inner helices are straight and bundle together at the intracellular end to produce a narrow opening lined with hydrophobic amino-acid residues, the hydrophobic seal [30], that restricts the movement of K^+^ ions in KcsA structure (the closed state). In MthK, the inner helices are bent at a conserved glycine that acts as a hinge, creating an obstacle-free path to ion passage. This type of gating is widespread among K^+^ channels, where the glycine hinge is highly conserved [31], in particular in K_2P_ channels, which have hinge glycine residues in their TM2 and TM4 helices [22]. Evidence for the presence of such a functional intracellular gate has been recently obtained in the *Drosophila* KCNK0 K_2P_ channel [32] and has been speculated to mediate the gating of TASK-2 by intracellular pH [18].

A second type of gating is thought to occur by a sort of constriction of the selectivity filter, first recognized in the form of C-type inactivation [33], and is sensitive to mutations at residues neighboring the external entrance of the pore. C-type inactivation might correspond to the deformation of the selectivity filter of the KcsA channel that has been seen in structures at low K^+^ concentrations [25]. It appears that a decrease in occupancy of the selectivity filter by K^+^ ions leads to its partial collapse with the carbonyl oxygen atoms of the filter projecting obliquely rather than towards the central axis of the conduction pathway. Opening and closing of the *Drosophila* KCNK0 channel have been demonstrated to entail extracellular K^+^ concentration-dependent C-type inactivation [34]. A similar mechanism has been proposed for pH_o_-gating of TASK-1 [35], TREK-1 [12] and TREK-2 [11].

A further type of gate mechanism, the ball-and-chain inactivation, has not been reported for K_2P_ channels.

### Validation of R224 as the pH_o_-sensor mediating the gating of TASK-2 channel

A set of five extracellular charged residues in the TM1-P1 extracellular linker of TASK-2 had been implicated as mediating channel response to alkalinisation [19]. This concept was, however, put in doubt with the observation that a quintuple mutant of TASK-2 neutralising all amino acids in the purported sensor maintained pH_o_-sensitivity [22]. We proposed an alternative mechanism for pH_o_-gating of TASK-2 in which a single arginine residue (R224) located near the second pore domain is responsible for sensing extracellular pH [20]. Based on molecular simulations, this study revealed a hydrophobic environment for R224, which is located at the outermost portion of TM4 near the pore region, for the titration of this residue, with a pK_a_ markedly shifted with respect to its free-solution value.

We put forth that the effect of R224 was exerted via an electrostatic effect on the pore that, in a manner that could only be speculated upon, prevented K^+^ permeation by promoting a blocked state [20]. We reasoned that if R224 is genuinely a pH_o_ sensor and by itself is capable of interfering with K^+^ permeability in its charged form, it should be possible to transfer this ability by transplantation into a channel that does not possess a charged residue at the equivalent position. We chose for this test the pH_o_-insensitive H98N mutant of TASK-3 [9], [10]. The location homologous to TASK-2 R224 residue corresponds to V221 in TASK-3, and its mutation to histidine led to pore collapse, as evidenced by a marked decrease in K^+^/Na^+^ permeability ratio and low currents in low K^+^ medium. Currents could be restored in high, symmetrical K^+^ allowing experiments that show that the TASK-2-type pH_o_-sensor transplantation led to restitution of pH_o_-gating. This successful transplantation experiment suggests that R224 is necessary and sufficient to confer pH_o_-gating to the TASK-2 channel, thus validating the identity of the sensor beyond reasonable doubt.

### One charged R224 residue out of two in the dimer is sufficient to gate TASK-2 channels by pH_o_

A concatenated channel approach was used to test whether both pH_o_-sensors are needed to gate the TASK-2 open and close. This type of approach has been used successfully in the study of multimeric functional proteins, in particular to explore subunit stoichiometry and other properties using TASK-1 channels [5]. Fully functional channels were obtained here by concatenating two TASK-2 channels. The currents observed in concatenated WT-WT channels did not differ from those obtained from their non-concatenated congeners. Also the pH_o_-dependence in concatenated channels did not differ from that of the normal channels. More interestingly, concatenated constructs carrying a single R224A mutation also behaved as WT monomeric channels. This clearly indicates that one changed arginine sensor is sufficient to lead to channel closure and that neutralisation of both sensors is necessary for channel opening. The fact that WT pH_o_-dependence is observed in WT-R224A or R224A-WT constructs also argues in favour of a lack of interaction between sensors.

Is the hypothesis that one charged pH_o_-sensor is sufficient to maintain channels closed and that neutralisation of both is necessary for activation supported by the energetics of the underlying process of permeation of K^+^ ions in the selectivity filter of TASK-2? To address this question we used adaptive biasing force (ABF) calculations [24] on a homology model of TASK-2 [20]. A major challenge of computational approaches consists in modeling rare events occurring in biological systems, which span time scales that are not amenable to classical molecular dynamics simulations. The difficulty in modeling these rare events is inherently rooted in the overwhelming number of degrees of freedom described explicitly in such statistical simulations, which is conducive of entrapment in local free-energy minima. Provided that a reasonable construct of the reaction coordinate underlying the process of interest can be inferred, the ABF method accelerates sampling and maps accurately the free-energy landscape through cancellation of the local free-energy derivative by the average force exerted along the model reaction coordinate.

Use of ABF here identified the selectivity filter K^+^-binding sites (S_0_–S_2_) found in all K^+^ channels investigated structurally [25], [26]. The study revealed a free-energy profile consistent with a high rate of ion translocation along the channel for a system with both R224 sensors in the neutral form. The channel with both R224 sensors positively charged exhibited high free-energy barriers for the S_0_-S_1_ and the S_1_-S_2_ transitions, anticipated to thwart translocation. Conversely, the result obtained for channels containing one neutralised R224 and one R224A mutated subunit had again low free-energy barriers, as expected for an active channel. Yet, charging the single available R224 residue in the channel led to a free-energy landscape featuring high barriers akin to the doubly charged WT-WT channel. In summary, the free-energy barriers seen under active channel conditions, with R224 sensors neutral in both subunits or the neutral-R224-A224 condition, are in rough agreement with those reported previously for KcsA and are found to be consistent with a high-rate translocation of the ions in the channel (24). In those instances where either one or two R224 sensors are charged, high S_0_-S_1_ and S_1_-S_2_ barriers arise and are expected to impede ion translocation along the selectivity filter.

### Mechanism for pH_o_-promoted gating at the pore of TASK-2

Of the K_2P_ channels gated by extracellular pH, TASK-1 and TASK-3 belong to the same branch of the superfamily and share a histidine residue, namely H98, located immediately above the GYG motif, which acts as a pH_o_-sensor in these channels [5], [9], [10]. Structural modelling and molecular dynamics studies of TASK-1 suggest that the neutral pH_o_-sensing histidine residue is buried behind the selectivity filter but switches to an extracellular-facing conformation upon protonation [35], [36]. This switch alters the stability of the selectivity filter turning carbonyl oxygen atoms away from the conduction pathway and, therefore, weakening markedly K^+^ ion interactions with the S_0_ and S_1_ sites [35], which is altogether consistent with pore collapse. Human TREK-1, although lacking the equivalent of pH_o_-sensing H98 of TASK-1, is also gated closed by extracellular acidification [12]. The sensing of pH_o_ in TREK-1 is mediated by entirely different sensors, namely histidine residues 87 and 141 located in the extracellular TM1-P1 loop. Interestingly, acidification-mediated inhibition of TREK-1 is accompanied by marked decreases in K^+^/Na^+^ permeability ratio strongly, hence suggesting that a collapse of the pore is responsible for channel closure [12].

Activation of TASK-2 by extracellular alkalinization is mediated by neutralization of R224 located near the second pore domain [20] and involves changes in open probability (P_o_) without affecting single channel conductance [8], [20], [21]. We proposed that, in the protonated form, R224 might decrease occupancy of the selectivity filter by K^+^, thus creating a blocked state, a situation relieved by neutralization of R224 at alkaline pH_o_ [20], [22]. This pH_o_-dependent gating of TASK-2 at the selectivity filter was speculated to occur by the type of occupancy-related changes in the pore structure revealed by molecular-dynamics simulations [37], as it is highly dependent upon [K^+^]_o_ [18]. These considerations would put pH_o_-gating at the pore in a similar category as that discussed above for TASK-1 and TREK-1. Decomposition of the free-energy profiles delineating ion permeation in the selectivity filter into VdW and electrostatic contributions, together with analysis of oxygen-chelation of K^+^ ions within the channel, however, argue for a completely different mechanism of gating.

The analysis revealed VdW repulsions that are compensated for by favourable electrostatic interactions along the selectivity filter in the doubly neutral sensor, “active” channel. In the doubly charged system, on the other hand, the opposite occurs, with negative values for the VdW contribution to free energy and strong electrostatic repulsion as expected from the two charged arginine residues. The large electrostatic contribution of the charged sensors in the in the R224^+^-R224^+^ system is compensated for by favourable short-range interactions between the selectivity filter oxygens and the permeant ions. Perhaps counter-intuitively so, a greater level of oxygen-chelation, particularly for carbonyl coordination at S_1_, occurs in the R224^+^-R224^+^ compared to the R224^0^-R224^0^ condition. This favourable vdW contribution reflects an increased flexibility of the selectivity filter that maximises the number ligands coordinating the ion. Consequently, the height of the energy barriers is increased owing to the high cost of dissociating the selectivity filter K^+^ ions from their carbonyl and water ligands. The results indeed suggest that the higher S_0_-S_1_ and S_1_-S2 energy barriers in the R224^+^-R224^+^ condition compared with the neutral system, favour the trapping of the K^+^ ions within the selectivity filter as the mechanism for blockade at acid pH_o_.

Our study provides no evidence for the type of pore collapse revealed in the molecular-dynamics study of TASK-1 [35], or the loss of K^+^ binding at S_2_ shown to take place in crystal structures of C-type inactivated KcsA channels [38]. Furthermore, the loss of K^+^ over Na^+^ selectivity observed when inhibiting TREK-1 by acidification [12] is also not present in TASK-2, as demonstrated in the original pH_o_-dependence description of this channel [8] and confirmed by us here. We conclude on the basis of our analysis that charging of pH_o_ sensors in TASK-2 is accompanied by channel closure by a process in which the permeating ion is trapped at binding site S_1_ and/or S_2_. This is associated with heightened barriers for ion translocation and does not entail a collapse of the selectivity filter.

## Methods

### Constructs

*Mus musculus* TASK-2 (GenBank accession N° AF319542) plasmid was previously obtained from mouse kidney. *Cavia porcellus* TASK-3 (GenBank accession N° AF212827), and its mutant TASK-3-H98N, were provided by Dr. Jürgen Daut (Marburg University, Germany). Mus musculus TASK-1 (originally named cTBAK-1, GenBank accession N° AB008537.1) was obtained from Dr. Donghee Kim (Rosalind Franklin University, Chicago). DNAs were subcloned into the pCR3.1 vector. Mutants and tandem constructs were generated using PCR with *Pfu* DNA polymerase by standard protocols. The PCR-amplified regions of all the constructs were confirmed by DNA sequencing.

Tandem dimer constructs of TASK-2, were generated by insertion of *Xba*I restriction sites to facilitate the concatenation. This insertion resulted in deletion of 148 amino acids in C-terminal of first monomer and the inclusion of L355 and D356 residues between the final amino acid of the first monomer (V354) and the initial M1 of the second monomer. The deletion became necessary to obtain reliable expression of the tandem constructs.

### Electrophysiological assays

Transient transfections were done in HEK-293 cells [39] as described [40]. Cells were transferred to the stage of a microscope for study, where they were continuously superfused with a bathing solution containing (in mM): 67.5 Na_2_SO_4_, 4 KCl, 1 K gluconate, 2 CaCl_2_, 1 MgCl_2_, 105 sucrose, and 10 Hepes/Tris (pH 7.5) (standard solution). The high K^+^ solution was obtained by equimolar replacement of Na^+^ by K^+^. The pipette solution contained (in mM):140 KCl, 1 MgCl_2_, 10 EGTA, 1 Na_3_ATP, 0.1 GTP, and 10 Hepes (pH 7.4). The HEPES buffer was replaced by MES in acidic solutions (pH 5.0–6.5) with AMPSO (8.5–9.0) and by CAPS in alkali solutions (pH 9.5–11.0). Standard whole-cell, patch-clamp recordings were performed as described elsewhere [40], [41]. Potentials were corrected for liquid junction shifts [42]. Acquisition and analysis was done with Clampfit 9.0 (Axon Instruments, Foster City, CA, USA). Acquisition was at 50 kHz with filter at 10 kHz (4-pole Bessel). Further data analysis was done using the curve fitting features of SigmaPlot v. 11 (Systat Software Inc., San José, CA, USA).

### Calculations

The effect of pH on currents was evaluated by plotting current (I) measured at a membrane potential of 0 mV, against extracellular [H^+^]. Fitting of a Hill equation to the data was done for each individual experiment. The parameters are defined in the following equation: I = I_min_ +(I_max_-I_min_)/(1+([H^+^]/*K_1/2_*)^nH^) (Eq. 1). For graphical representation, average ± S.E.M. I/I_max_ values were obtained from individual experiments and plotted together with curves constructed with average parameters obtained from the individual fits. Fits were done using the Marquardt-Levenberg algorithm as implemented in the SigmaPlot software.

### Molecular Models

The molecular model of the pore of TASK-2 channel was built with the program MODELLER version 9 [43], as published in Niemeyer et al. [20], using as a template the crystallographic structure of the Kv1.2 potassium channel (PDB code 2A79). This template was chosen because it is a potassium channel, for which the three-dimensional structure of its open conformation is known. Intra- and extracellular loops were not modeled.

Four models were built. The first one, the WT model, features two R224 residues in their neutral state. The second one is its charged counterpart, featuring two positively charged R224 residues. In the third model, one of the R224 residues was mutated into alanine, whilst the other residue remained charged. In the fourth model the latter arginine residue was neutralized.

The longitudinal axis of TASK-2 WT channel was collinear to the *z*-axis of Cartesian space. The membrane protein was immersed in a fully hydrated palmitoyloleylphosphatidylcholine (POPC) bilayer. The initial dimensions of the complete molecular assembly were approximately 102×101×86 Å^3^. Periodic boundary conditions were utilized. Construction of the molecular assemblies was performed with the VMD program [44].

### Molecular-dynamics simulations

All molecular dynamics simulations were performed in the isobaric-isothermal ensemble, using the program NAMD [45] with the CHARMM force field and the TIP3P water model [46]. The temperature was maintained at 310 K, using Langevin dynamics with a damping coefficient of 1 ps^−^1^^. The pressure was fixed at 1 atm. by means of the Langevin piston method. The equations of motion were integrated employing the Verlet r-RESPA algorithm [47], with a time step of 1 fs. Short-range, van der Waals interactions were truncated smoothly with a 12 Å spherical cutoff. Long-range electrostatic forces were taken into account by means of the particle-mesh Ewald approach [48]. Covalent bonds between heavy and hydrogen atoms were constrained to their equilibrium length using the RATTLE algorithm [49].

After appropriate energy minimization, the system was equilibrated in the canonical ensemble at 310 K for 500 ps, and then in the isobaric-isothermal ensemble for 1 ns, using harmonic restraints of 0.5 kcal/mol/Å^2^ applied to alpha carbon atoms.

### Free-energy calculations

To measure the free energy associated to the translocation of the ion along the selectivity filter, a multi-ion configuration was used. Three ions denoted K1, K2 and K3, were placed at sites S_0_, S_2_ and S_4_, respectively. Simultaneously, three water molecules (W) were placed at binding sites S_1_ and S_3_, and on the extracellular side of site S_0_. The distance between each member of the chain W–K^+^–W–K^+^–W–K^+^ was restrained gently to 3.5 Å with a force constant of 5 kcal/mol/Å^2^. This approach was adopted to improve the exploration of the surrogate reaction coordinate.

To explore the free-energy landscape delineating ion transport through the TASK-2 pore, the reaction coordinate was modeled by a one-dimensional order parameter, *ξ*, corresponding to the distance separating ion K2 from the center of mass formed by sixteen alpha carbons of the selectivity filter, projected onto the longitudinal axis *z*. Variation of the free energy, Δ*G*, with *ξ* was determined using the adaptive biasing force (ABF) method [24], which relies on the integration of the average force acting along *ξ* measured from unconstrained MD simulations. The biasing force estimated locally is then applied to the system and yields a Hamiltonian in which no average force acts along *ξ*. Exploration of *ξ* is governed by the self-diffusion properties of the system, which, assuming reasonable decoupling from other slow degrees of freedom, guarantees uniform sampling [50]. The instantaneous force acting along *ξ* was accrued in small bins, 0.1 Å wide. The reaction pathway was divided into three consecutive, non-overlapping, 2-Å wide windows. In each window, up to 20 ns of MD trajectory were generated, which corresponds for each of the four channels, viz. R224^+^-R224^+^, R224°-R224°, R224^+^-R224A and R224°-R224A, to a total sampling of 60 ns, and an aggregated simulation time of 240 ns for the present study. The standard error associated to the free-energy difference was estimated using the formula of Rodríguez-Gómez [51].

## Supporting Information

Figure S1 **Apparent changes in K^+^/Na^+^ selectivity in TASK-3 and -1 accompanying inhibition by extracellular acidification.** Current-voltage relations are shown for cells expressing TASK-3 or TASK-1 at two extracellular pH values chosen to yield respectively ∼90 and ∼15% of the maximal activation for each one of the channels as judged from complete activity vs. pH curves. The intracellular solution contained 140 mM K^+^, whilst extracellular bath had 135 mM Na^+^ and 5 mM K^+^. The current-voltage relations were taken from 250 ms-voltage ramps given from the most hyperpolarised voltage. It can be seen that for TASK-3 and, more obviously, for TASK-1, inhibition was accompanied by a displacement of the reversal potential (E_rev_) to a more depolarised position on the voltage axis. On the right graphs summarise average E_rev_ measurements confirming this impression. The results are means ± SEMs, n = 7 and 6 experiments for TASK-3 and TASK-1 respectively. E_rev_ became significantly (as analysed by paired t-test) more positive upon acidification. The results for TASK-3 and TASK1 are similar to that found for TREK-1, and which has been interpreted to imply that gating at the selectivity filter by extracellular pH occurs by a “collapse of the external pore gate, similar to the C-type inactivation of voltage-gated potassium channels” [12]. Use of this criterion, and comparison with the result for TASK-2 shown in Figure 2, it would appear that the same mechanism does not to apply to the gating of TASK-2 by extracellular protons.
(TIF)

Figure S2 **Potassium currents mediated by concatenated constructs of WT and pH_o_-insensitive TASK-2 channels**. Currents recorded were elicited by transfection of concatenated constructs formed by joining two TASK-2 (WT-WT), two TASK-2-R224A (R224A-R224A) channels or mixed tandem constructs of the form TASK-2/TASK-2-R224 (WT-R224A) or TASK-2-R224A/TASK-2 (R224A-WT) into HEK-293 cells. Measurements were done in the whole-cell recording mode of the patch-clamp technique, using the same voltage protocol as in Fig. 1*a* (for *a*, *c*, *e* and *g*) or Fig. 1*b* (for *b*, *d*, *f* and *h*) of the paper. The intracellular solution contained 140 mM K^+^. The extracellular medium had 135 mM Na^+^ and 5 mM K^+^ in *a*, *c*, *e* and *g*. In *b*, *d*, *f* and *h*, extracellular Na^+^ was replaced by an equimolar amount of K^+^.
(TIF)
