# Supplementary figures and images for: Gating of a pH-Sensitive K2P Potassium Channel by an Electrostatic Effect of Basic Sensor Residues on the Selectivity Filter
Source: PLoS One. 2011 Jan 25;6(1):e16141. doi: 10.1371/journal.pone.0016141 (PMC3026807; doi:10.1371/journal.pone.0016141)

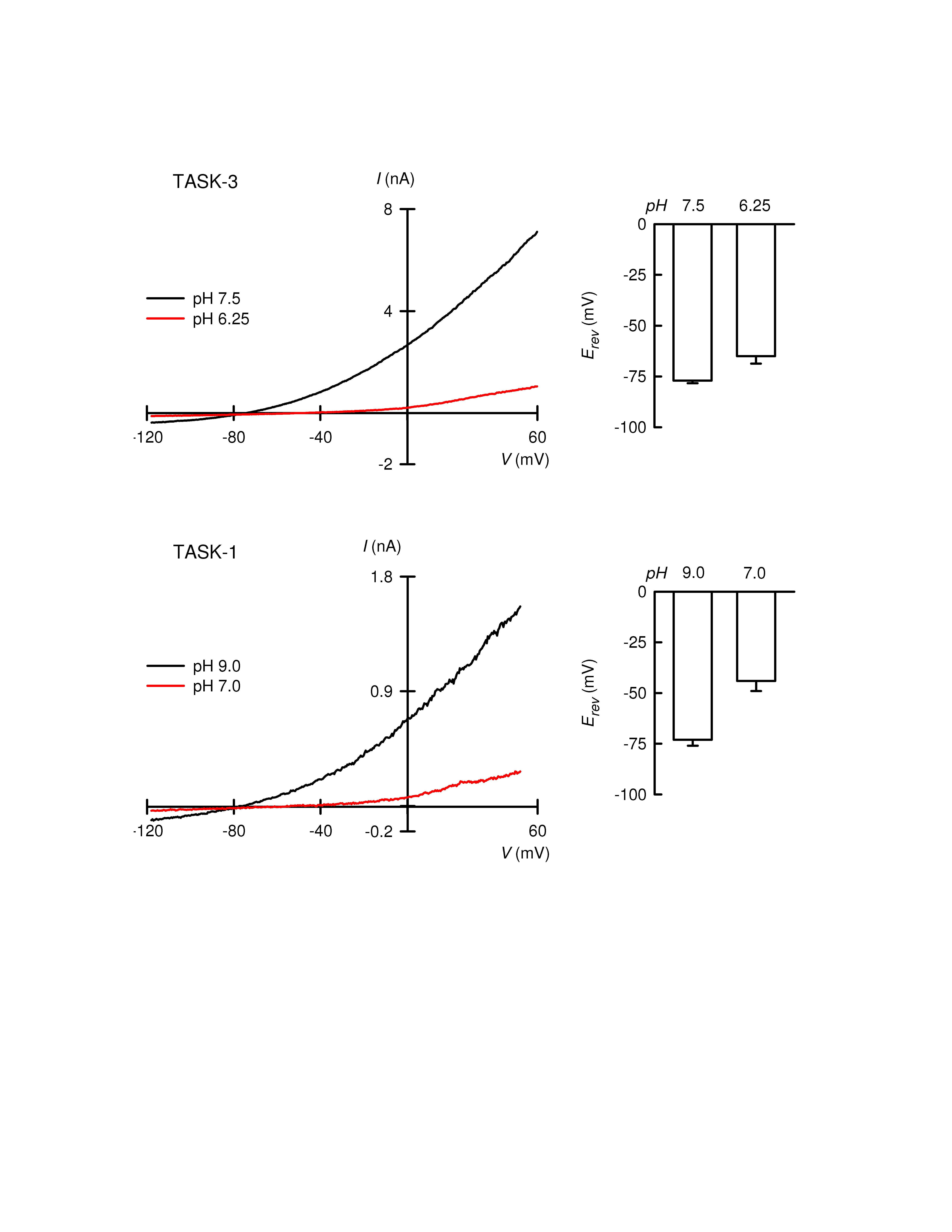

Supplement: Figure S1 — Apparent changes in K+/Na+ selectivity in TASK-3 and -1 accompanying inhibition by extracellular acidification. Current-voltage relations are shown for cells expressing TASK-3 or TASK-1 at two extracellular pH values chosen to yield respectively ∼90 and ∼15% of the maximal activation for each one of the channels as judged from complete activity vs. pH curves. The intracellular solution contained 140 mM K+, whilst extracellular bath had 135 mM Na+ and 5 mM K+. The current-voltage relations were taken from 250 ms-voltage ramps given from the most hyperpolarised voltage. It can be seen that for TASK-3 and, more obviously, for TASK-1, inhibition was accompanied by a displacement of the reversal potential (Erev) to a more depolarised position on the voltage axis. On the right graphs summarise average Erev measurements confirming this impression. The results are means ± SEMs, n = 7 and 6 experiments for TASK-3 and TASK-1 respectively. Erev became significantly (as analysed by paired t-test) more positive upon acidification. The results for TASK-3 and TASK1 are similar to that found for TREK-1, and which has been interpreted to imply that gating at the selectivity filter by extracellular pH occurs by a “collapse of the external pore gate, similar to the C-type inactivation of voltage-gated potassium channels” [12]. Use of this criterion, and comparison with the result for TASK-2 shown in Figure 2, it would appear that the same mechanism does not to apply to the gating of TASK-2 by extracellular protons. (TIF) [file pone.0016141.s001.tif]

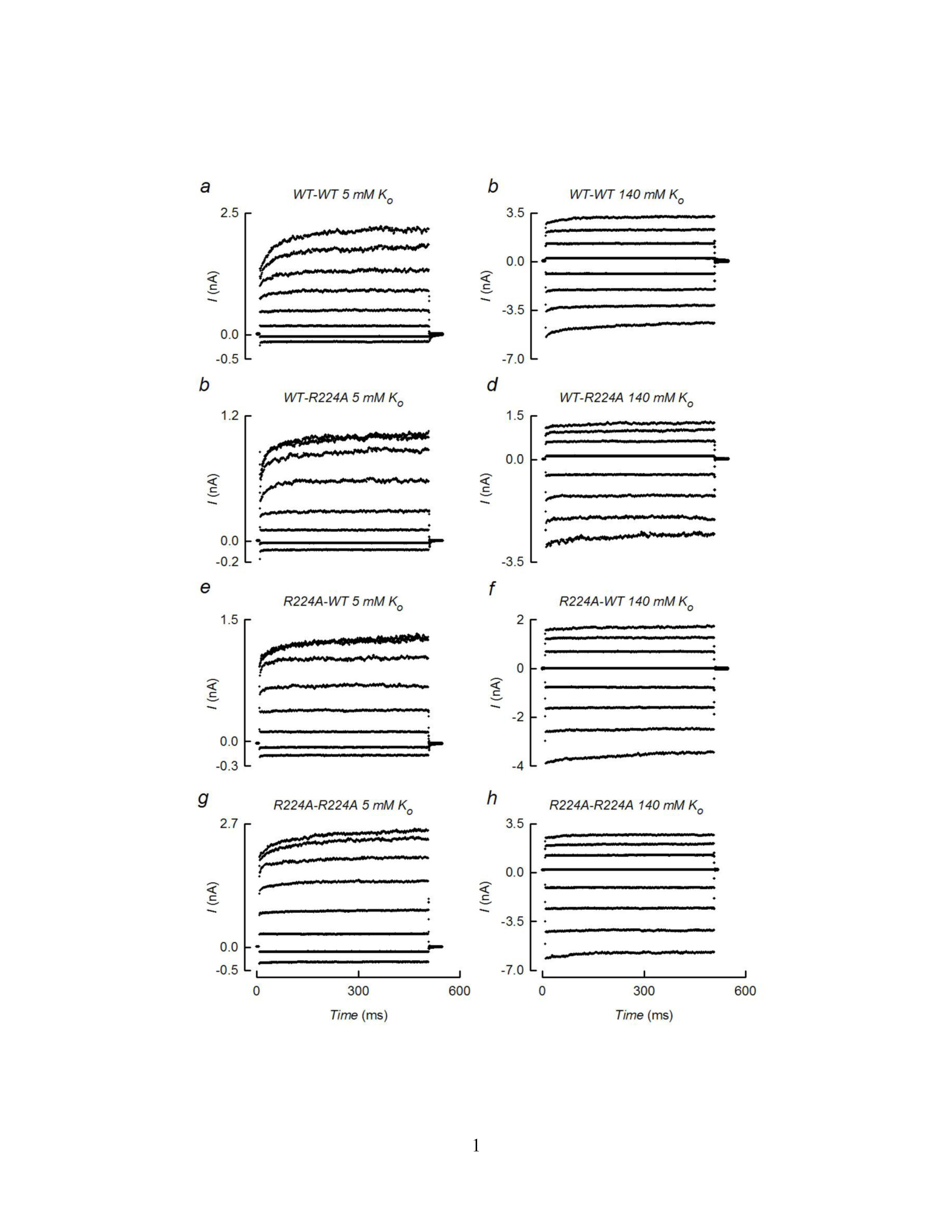

Supplement: Figure S2 — Potassium currents mediated by concatenated constructs of WT and pHo-insensitive TASK-2 channels. Currents recorded were elicited by transfection of concatenated constructs formed by joining two TASK-2 (WT-WT), two TASK-2-R224A (R224A-R224A) channels or mixed tandem constructs of the form TASK-2/TASK-2-R224 (WT-R224A) or TASK-2-R224A/TASK-2 (R224A-WT) into HEK-293 cells. Measurements were done in the whole-cell recording mode of the patch-clamp technique, using the same voltage protocol as in Fig. 1a (for a, c, e and g) or Fig. 1b (for b, d, f and h) of the paper. The intracellular solution contained 140 mM K+. The extracellular medium had 135 mM Na+ and 5 mM K+ in a, c, e and g. In b, d, f and h, extracellular Na+ was replaced by an equimolar amount of K+. (TIF) [file pone.0016141.s002.tif]
